# Myelin Basic Protein-Induced Production of Tumor Necrosis Factor-α and Interleukin-6, and Presentation of the Immunodominant Peptide MBP85-99 by B Cells from Patients with Relapsing-Remitting Multiple Sclerosis

**DOI:** 10.1371/journal.pone.0146971

**Published:** 2016-01-12

**Authors:** Claus H. Nielsen, Lars Börnsen, Finn Sellebjerg, Marie K. Brimnes

**Affiliations:** 1 Institute for Inflammation Research, Center for Rheumatology and Spine Diseases, Copenhagen University Hospital Rigshospitalet, Copenhagen, Denmark; 2 Department of Neurology, Copenhagen University Hospital Rigshospitalet, Danish Multiple Sclerosis Center, Copenhagen, Denmark; Medical University of Innsbruck, AUSTRIA

## Abstract

B cells are involved in driving relapsing-remitting multiple sclerosis (RRMS), as demonstrated by the positive effect of therapeutic B-cell depletion. Aside from producing antibodies, B cells are efficient antigen-presenting and cytokine-secreting cells. Diverse polyclonal stimuli have been used to study cytokine production by B cells, but here we used the physiologically relevant self-antigen myelin basic protein (MBP) to stimulate B cells from untreated patients with RRMS and healthy donors. Moreover, we took advantage of the unique ability of the monoclonal antibody MK16 to recognize the immunodominant peptide MBP85-99 presented on HLA-DR15, and used it as a probe to directly study B-cell presentation of self-antigenic peptide. The proportions of B cells producing TNF-α or IL-6 after stimulation with MBP were higher in RRMS patients than in healthy donors, indicating a pro-inflammatory profile for self-reactive patient B cells. In contrast, polyclonal stimulation with PMA + ionomycin and MBP revealed no difference in cytokine profile between B cells from RRMS patients and healthy donors. Expanded disability status scale (EDSS) as well as multiple sclerosis severity score (MSSS) correlated with reduced ability of B cells to produce IL-10 after stimulation with MBP, indicative of diminished B-cell immune regulatory function in patients with the most severe disease. Moreover, EDSS correlated positively with the frequencies of TNF-α, IL-6 and IL-10 producing B cells after polyclonal stimulation. Patient-derived, IL-10-producing B cells presented MBP85-99 poorly, as did IL-6-producing B cells, particulary in the healthy donor group. B cells from MS patients thus present antigen to T cells in a pro-inflammatory context. These findings contribute to understanding the therapeutic effects of B-cell depletion in human autoimmune diseases, including MS.

## Introduction

Multiple sclerosis (MS) is an autoimmune, demyelinating disease affecting the central nervous system [1]. Although MS is considered a T-cell mediated disease [1], accumulating data suggest that B cells also participate in disease development [2–5]. Most convincing are clinical studies in which MS patients received the B-cell depleting anti-CD20 antibodies rituximab or ocrelizumab [6–9]. The antibody-producing plasma cells are not targeted directly by rituximab, and total immunoglobulin levels in cerebrospinal fluid or oligoclonal bands are not significantly affected by this treatment [10,11]. Nevertheless, the number of lesions and relapses in relapsing-remitting MS (RRMS) patients is significantly reduced during B-cell depletion therapy [6,7], suggesting that B cells play a role in RRMS pathology by virtue of their antigen-presenting capacity [12], or by virtue of their ability to produce cytokines [13,14].

B cells can capture antigen, even at low concentrations, via their B-cell receptor (BCR), and up-concentrate, internalize, process and present the antigen efficiently [12]. We [15] and others [16,17] have demonstrated that also non-specific B cells can capture and present antigens in a complement-dependent manner, which vastly increases the pool of B cells available for antigen presentation. Studies in experimental autoimmune encephalomyelitis (EAE), the primary mouse model of RRMS, demonstrate that B cells play a significant role as antigen-presenting cells (APCs), participating in re-activation of auto-reactive T cells in the central nervous system [18] and probably also in lymph nodes [16].

Cytokines produced by B cells comprise among others interleukin(IL)-2, IL-4, IL-6, IL-10, interferon (IFN)-α, IFN-γ, TNF-α, TGF-β and IL-17 (for review see [14]). These cytokines affect different cell types, and both regulatory and pathogenic effects of B-cell cytokines have been reported. For example, IL-10-producing B cells are known to protect against development of EAE [19,20], while IL-6-producing B cells aggravate EAE [21]. B cells from patients with RRMS secrete more IL-6 and appear at higher frequencies after polyclonal stimulation than B cells from healthy donors [21,22]. Some investigators have also found increased secretion of lymphotoxin (LT) and TNF-α by B cells from RRMS patients stimulated polyclonally [5,22], while others found no increased production of these pro-inflammatory cytokines [23]. Several authors have reported an impaired ability of B cells from RRMS patients to secrete IL-10 after polyclonal stimulation [22,23]. B cells from RRMS patients therefore appear to represent a more pro-inflammatory phenotype than B cells from healthy donors, when subjected to non-specific stimuli.

Antigen presentation and cytokine production by B cells may occur simultaneously and may shape the resulting T-cell response, leading to activation of T cells with a pro-inflammatory phenotype. For example, B-cell derived IL-6 and IFN-γ are important for polarizing effector T-cell responses into Th17 and Th1 responses in *Salmonella enterica*-infected mice [24]. In EAE, lack of IL-6-production by B cells leads to an impaired Th17 response *in vivo*, demonstrating the importance of IL-6 in driving the pro-inflammatory effector T-cell response [21].

In general, polyclonal stimulation with phorbol 12-myristate 13-acetate (PMA) + ionomycin, CpG or BCR cross-linking has been used to study cytokine production by B cells. While this approach may show the potential of the entire B-cell pool to differentiate into cytokine-producing cells, it does not reflect the more physiological situation where B cells may be stimulated clonally with self-antigens and receive help from antigen-specific T-helper cells. Only a few studies have addressed antigen-specific induction of cytokine production by B cells, and, to our knowledge, only two of these in humans have used stimulation with disease-relevant self-antigens [25,26].

Usually, antigen presentation by B cells is studied by examination of T-cell responses induced by antigen-pulsed B cells. However, this approach does not reveal antigen presentation that leads to anergization of T cells, as might be expected for presentation of self-antigens in healthy individuals, and it does not show the antigen presentation occurring in a blood- or lymph node-like environment, where monocyte-derived cells are abundant and able to generate a pro- or anti-inflammatory environment. In a cohort of healthy donors, we previously directly examined the B-cell uptake and presentation of the self-antigen myelin basic protein (MBP), a self-antigen considered to be involved in the pathogenesis of MS [15]. Here, we investigated the ability of B cells from MS patients to produce cytokines and present MBP85-99 peptide. Furthermore, we investigated whether these parameters correlate with disease severity.

## Materials and Methods

### Patients and healthy donors

Patients were recruited from the Department of Neurology, Rigshospitalet, Denmark. MS patients had been diagnosed according to the McDonald criteria [27]. Blood samples were collected in heparin-containing tubes (BD Biosciences, San Jose, CA) from 13 RRMS patients, 5 men and 8 women aged 36.7 ± 9.9 years (mean ± standard deviation). All patients were assessed for disease severity using the Multiple Sclerosis Severity Score (MSSS) [28] and the Kurtzke Expanded Disability Status Scale (EDSS) [29]. Both scales are measures of the severity of disease. The EDSS measures the degree of severity at a given time, and the MSSS adjusts the EDSS score according to disease duration, thus two patients with the same EDSS score but different disease durations will have different MSSS scores, and the patient who has the shortest duration of illness has the highest MSSS score. The study was approved by the Research Ethics Committees of the Capital Region of Denmark (# KF 01 314009) and conducted in accordance with the provisions of the Declaration of Helsinki. All participants gave written informed consent. 12 healthy blood donors, 4 men and 8 women, aged 41.0 ± 12.2 years (mean ± standard deviation) attending the Blood Bank at Copenhagen University Hospital Rigshospitalet served as controls. HLA-DR15-positive blood donors were used for studies of MK16 binding.

### Cells and serum

Peripheral blood mononuclear cells (PBMCs) were isolated by gradient centrifugation over LymphoPrep (Axis-Shield, Oslo, Norway). The cells were washed twice in phosphate buffered saline (GIBCO, Invitrogen, Carlsbad, CA) and were resuspended in Roswell Park Memorial Institute (RPMI) 1640 medium containing HEPES (Biological Industries Israel Beit-Haemek Ltd, Kibbutz Beit-Haemek, Israel), L-glutamine (GIBCO) and gentamicin (GIBCO). Cells were stored in liquid nitrogen before use. Serum from blood group AB donors (SIGMA-ALDRICH, St. Louis, MO) was used as source of normal human serum.

### HLA-typing

The single nucleotype polymorphism rs9271366, which tags the HLA-DRB1*15:*01* allele, was genotyped by TaqMan allelic discrimination PCR assay (Life Technologies Europe BV, Denmark) using predesigned primers and probes as previously described [30].

### Antigens and antibodies

Whole human MBP was purchased from HyTest Ltd. (Turku, Finland). The monoclonal antibody MK16, which recognizes MBP85-99 in the context of HLA-DRB1*15:01, was used as probe for antigen presentation [31]. The MK16 IgG1 antibody was affinity-purified by protein A from the supernatant of MK16-expressing Chinese hamster ovary cells grown in HAMS F-12 media (GIBCO) supplemented with 10% fetal calf serum (FCS; Biological Industries) and 0.8 mg/ml geneticin (Invitrogen, Carlsbad, CA). Antibodies used for flow cytometry were: PE-Cy7-streptavidin, PerCP-Cy5.5-anti-human CD19 (clone HIB19), PE-anti-human CD3 (clone UCHT1), APC-anti-human CD3 (clone UCHT1), PE-anti-human TNF-α (clone MAb11), FITC-anti-human IL-6 (clone AS12) (all from BD Biosciences) and APC-anti-human IL-10 (clone JES3-19F1)(Biolegend, San Diego, CA).

### Assessment of MBP presentation and intracellular cytokine staining

0.5x10^6^ PBMCs were incubated for 18 h at 37°C under 5% CO_2_ in RPMI-1640 containing 30% (v/v) serum from healthy blood group AB donors in a final volume of 200 μl with either: no stimulating antigen, 30 μg/ml MBP, or 30 μg/ml MBP plus cell stimulation cocktail containing PMA and ionomycin (500x diluted from stock; PMA 40.5uM and 670 μM ionomycin)(eBioscience, San Diego, CA). The cocktail was added during the last 4 h of culture. To block secretion of cytokines, 1 μl/ml of 1:5 diluted brefeldin A (1000x #555029 BD Biosciences), was added to all cultures during the last 4 h. Next, the cells were incubated with IgG for intravenous use (IVIg; CSL Behring, Bern, Switzerland) at a concentration of 6 mg/ml with 2% mouse serum (Statens Serum Institut, Copenhagen, Denmark) to block unspecific binding. Subsequently, MK16 was incubated at a concentration of 50 ng/ml for 30 min at 4°C in 2% FCS; antibodies against cell-surface markers were included in the same step. Following two washes, streptavidin-PE-Cy7 was incubated with the samples for 30 min at 4°C. For intracellular staining of cytokines, Cytofix/Cytoperm^™^ solution (BD Biosciences) was used according to the manufacturer’s instructions. The LIVE/DEAD^®^ Fixable Near-IR Dead Cell Stain Kit from Molecular Probes^®^ (Molecular Probes, Eugene, OR, USA) was used to discriminate between live and dead cells. First a live/dead cell gate was used to discriminate living cells from dead cells. Next, doublets were excluded based on FSC-A and FSC-W. Finally, B cells were identified as CD19 positive cells within the lymphocyte gate. Cells were analyzed on a FACS Canto flow cytometer (BD Biosciences), and data was analyzed using FlowJo v.X, (TreeStar, Inc, Ashland, OR).

### Statistics

Statistical analysis was performed using GraphPad Prism version 6 (GraphPad Software, La Jolla, CA). Comparisons between RRMS patients and healthy donors were performed using the two-tailed Mann Whitney U-test. Comparisons between non-stimulated and MBP-stimulated B cells were done using the Wilcoxon matched-pairs signed-rank test. Column statistics were calculated using the Wilcoxon signed-rank test. The non-parametric Spearman’s correlation test was used to analyze the association between cytokine positive B cells and EDSS or MSSS.

## Results

### MBP-induced cytokine-producing B cells

To study the ability of an MS-relevant self-antigen to stimulate cytokine production by B cells derived from RRMS patients and those derived from healthy donors, we determined the frequencies of B cells producing TNF-α, IL-6 or IL-10 before and after stimulation of PMBCs from these groups with MBP. The flow cytometric gating strategy is shown in S1 Fig.

**Fig 1:**
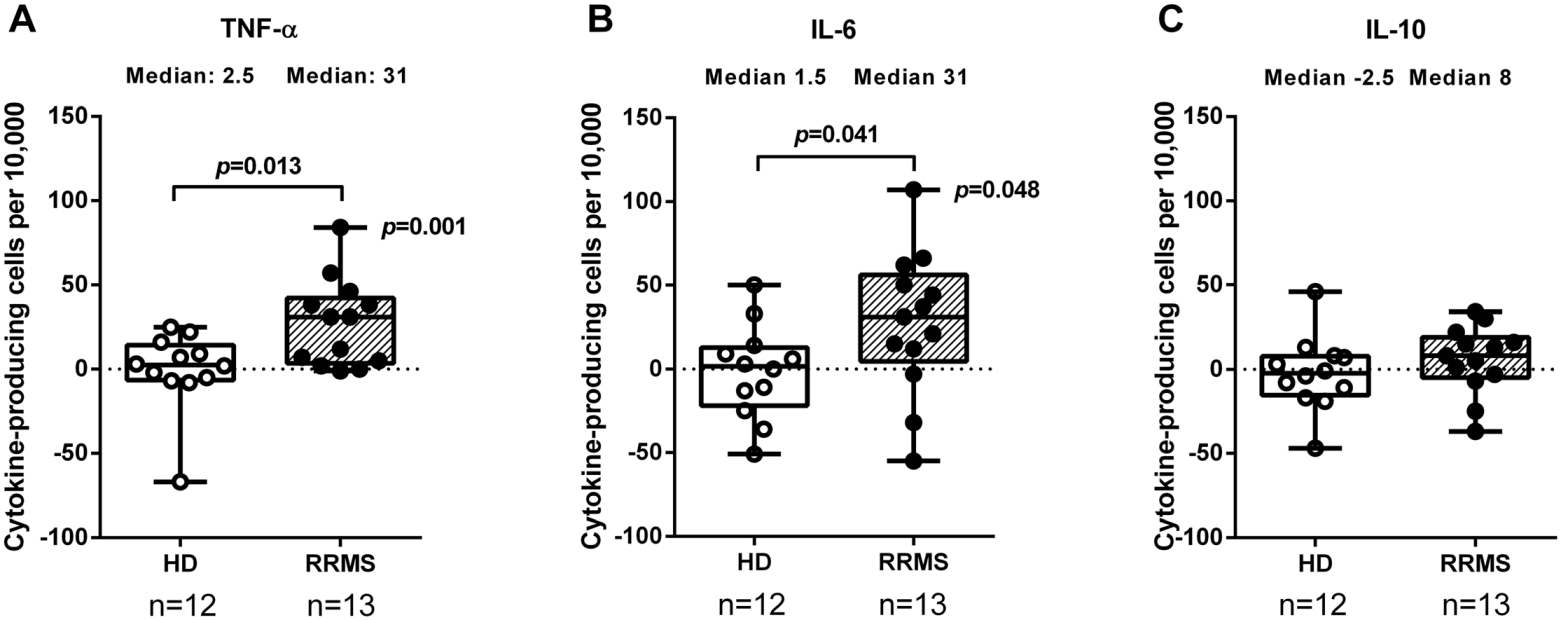
MBP-induced cytokine production by B cells. Mononuclear cells from 12 healthy donors (HD) and 13 patients with relapsing-remitting multiple sclerosis (RRMS) were stimulated with whole myelin basic protein (MBP) for 24 hours and stained intracellularly for (A) TNF-α, (B) IL-6, and (C) IL-10 before assessment by flow cytometry. The proportions of CD19+ B cells producing these cytokines are shown as median, interquartile range (box) and range (whiskers), adjusted for background (positive events in unstimulated cell cultures). In some cases these numbers were larger than in MBP-stimulated cultures, hence negative values. *p*-values indicate the probability of no difference between the groups (two-tailed Mann Whitney U-test) or from zero (Wilcoxon signed-rank test).

Stimulation with MBP increased the proportion of TNF-α-producing B cells and the proportion of IL-6 producing B cells from RRMS patients, while only minor changes were seen in the proportions of TNF-α- or IL-6-producing B cells from healthy donors (Fig 1A and 1B). MBP induced only few IL-10-producing B cells in both groups (Fig 1C). Raw values for all cytokine data are presented in S2 Fig.

**Fig 2:**
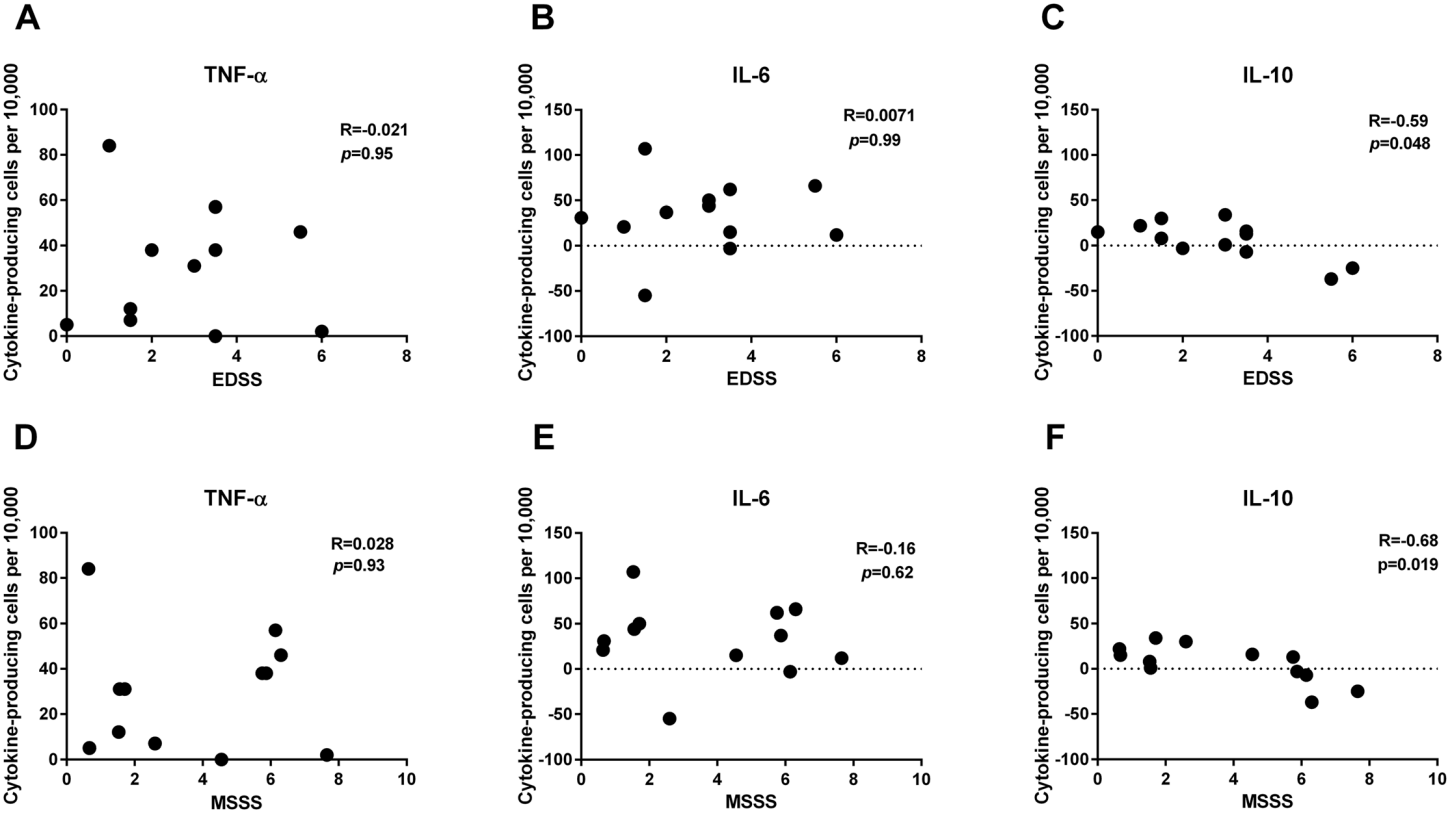
Association between MBP-induced cytokine production by B cells and disease severity. Mononuclear cells from 12 relapsing-remitting multiple sclerosis patients were stimulated with myelin basic protein (MBP) for 24 hours, stained for content of (A and D) TNF-α, (B and E) IL-6, or (C and F) IL-10, and assessed by flow cytometry. The proportion of cytokine-producing B cells adjusted for background (un-stimulated cells) is shown as a function of the Expanded Disability Status Scale (EDSS; upper row) and the Multiple Sclerosis Severity Score (MSSS; lower row)(both missing for one patient). Spearman’s correlation coefficient (R_S_) and the corresponding *p*-values are also shown.

The MBP-induced differentiation of cytokine-producing B cells depended on disease severity, as measured by both the MSSS score, a measure of disease severity corrected for disease duration, and the EDSS score (Fig 2). We did not observe any correlation between the frequency of IL-6-producing and TNF-α-producingf B cells and disease severity. However, for both the EDSS and MSSS scores, we found correlations with the frequency of IL-10-producing B cells. In the patients with the most severe disease, the ability of B cells to produce IL-10 was abrogated (Fig 2C and 2F).

### Maximum inducible frequency of cytokine-producing B cells

**Fig 3:**
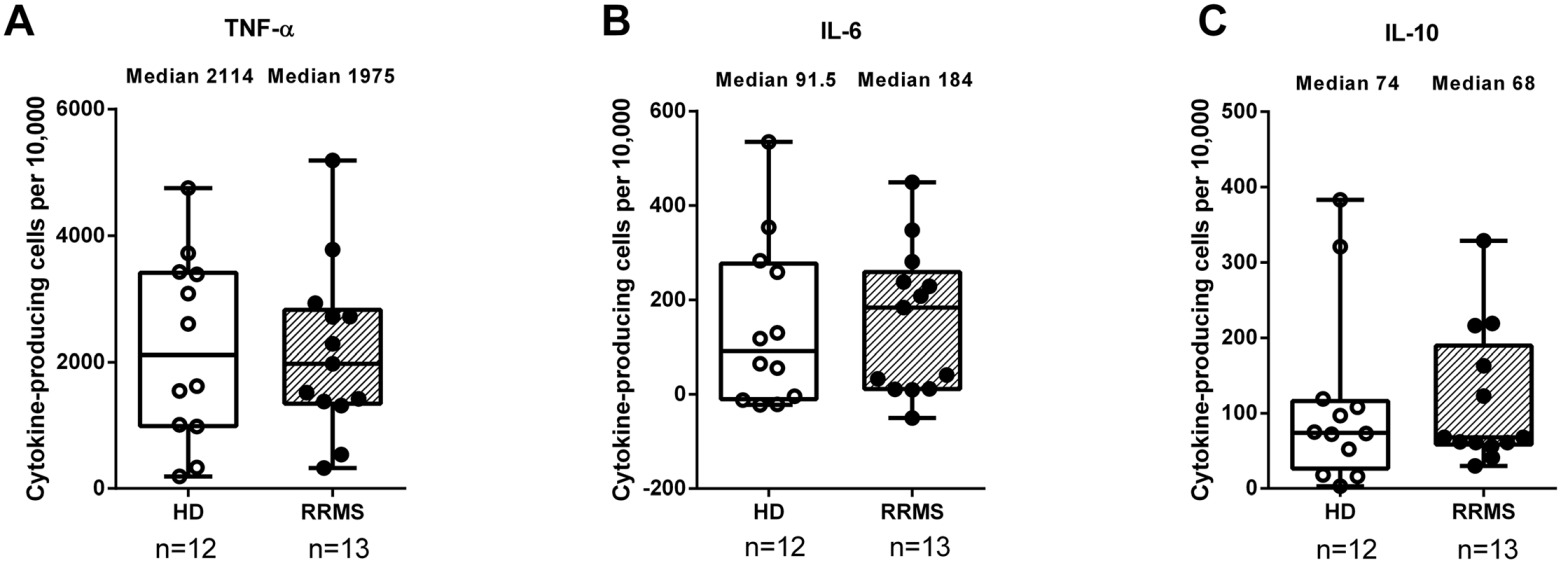
Proportions of cytokine-producing B cells after polyclonal stimulation. Mononuclear cells from 12 healthy donors (HD) and 13 relapsing-remitting multiple sclerosis (RRMS) patients were stimulated with myelin basic protein (MBP) for 24 hours, and with PMA + ionomycin for the last 4 hours of incubation. Cells were stained intracellularly with antibodies against (A) TNF-α, (B) IL-6 and (C) IL-10, and assessed by flow cytometry. Shown are the proportions of CD19+ B cells producing these cytokines; the corresponding values for unstimulated cell cultures have been subtracted. Box plots indicate median, interquartile range (box) and range (whiskers).

To induce maximum production of cytokines, cells were stimulated with PMA + ionomycin [32,33]. MBP was also added to enable analysis of the B cells’ ability to present the MBP-derived peptide MBP85-99 (see below). After this polyclonal stimulation, a large proportion of B cells from healthy donors and B cells from RRMS patients produced TNF-α, IL-6, or IL-10 (Fig 3). The frequencies did not differ between the groups.

**Fig 4:**
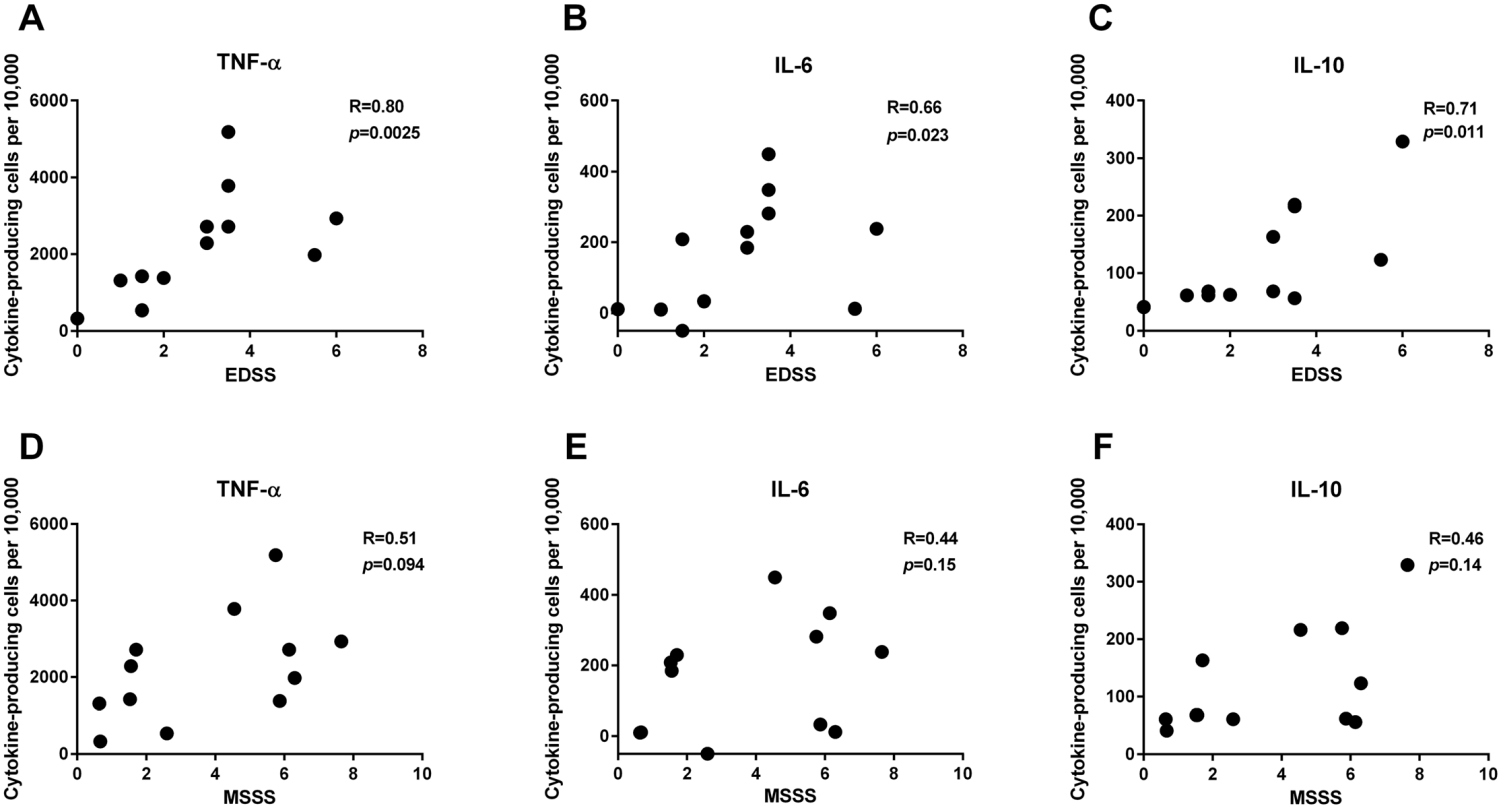
Association between polyclonally induced B-cell cytokine production and disease severity. Mononuclear cells from 13 patients with relapsing-remitting multiple sclerosis were stimulated with myelin basic protein (MBP) for 24 hours and PMA + ionomycin for the last 4 hours of incubation. Cells were stained for intracellular content of (A and D) TNF-α, (B and E) IL-6, and (C and F) IL-10 and assessed by flow cytometry. The proportions of CD19+ B cells producing these cytokine are shown as a function of the Expanded Disability Status Scale (EDSS; upper row) and the Multiple Sclerosis Severity Score (MSSS; lower row)(both missing for one patient). The corresponding values from unstimulated cultures have been subtracted. Spearman’s correlation coefficient, R_S_, and the corresponding *p*-values are also shown.

Interestingly, however, the proportion of cytokine-producing B cells induced by PMA + ionomycin in combination with MBP correlated with disease severity as measured by the EDSS score, as shown in Fig 4A–4C for TNF-α, IL-6, and IL-10. Similar findings were made for the MSSS score, although here there was no statistically significant correlation (Fig 4E and 4F).

### Presentation of MBP85-99 by B cells

**Fig 5:**
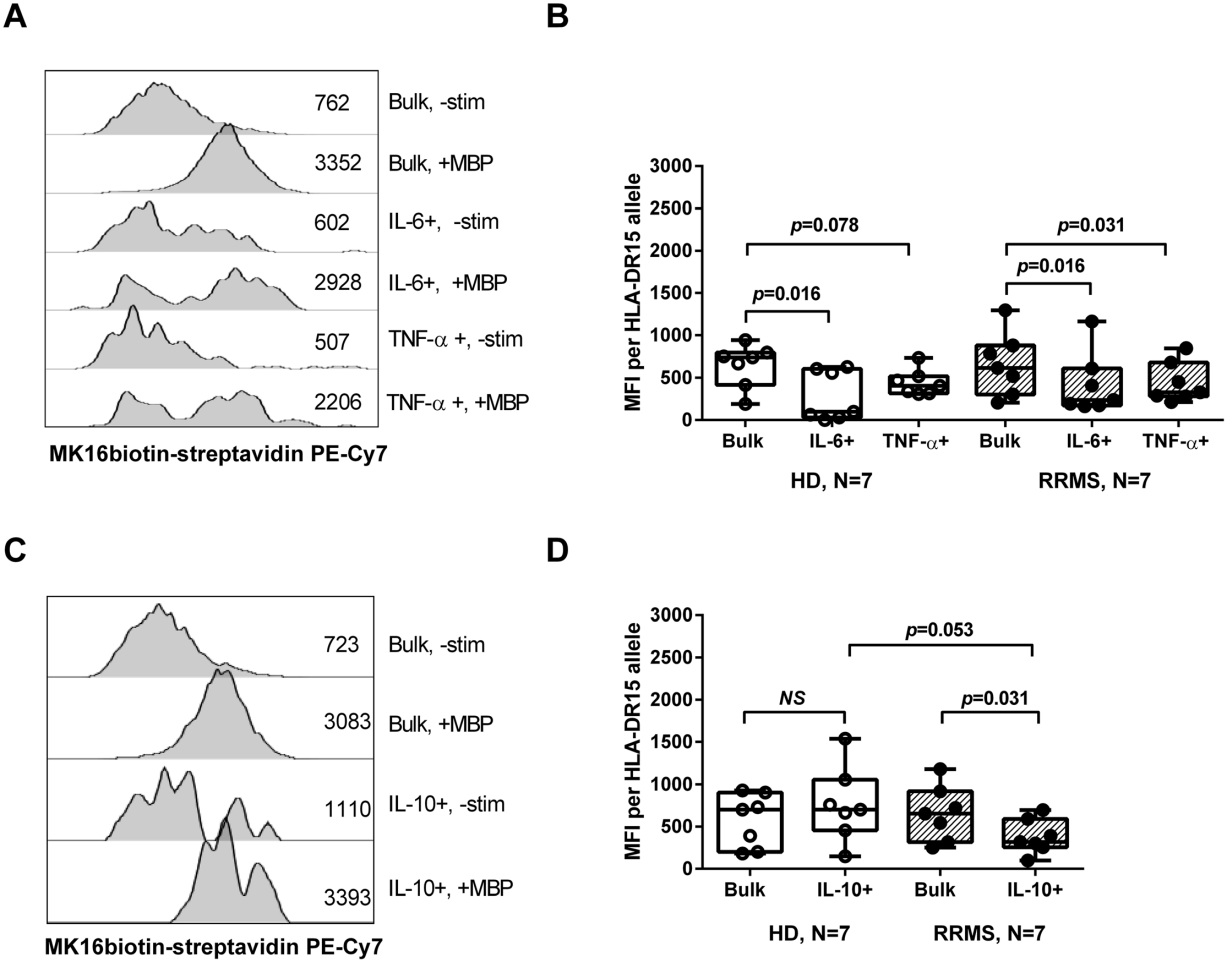
Presentation of MBP85-99 and cytokine production by HLA-DR15+ B cells. Mononuclear cells from 7 healthy donors (HD; all heterozygous for HLA-DR15) and 7 RRMS patients (2 homozygous, and 5 heterozygous for HLA-DR15) were either left unstimulated (-Stim), or were stimulated with whole myelin basic protein (MBP) for 24 hours. Cells were then stained with the mAb MK16, recognizing the MBP-derived peptide MBP85-99 presented on HLA-DR15. (A and C) Representative histogram plots showing MK16 binding to the total CD19+ B-cell pool (bulk) and the subsets of B cells producing IL-6, TNF-α, and IL-10. (B) Median fluorescence intensity (MFI) values of MK16 binding to bulk B cells and B cells producing IL-6, TNF-α, and (D) IL-10 after MBP stimulation are shown as median, interquartile range (box) and range (whiskers). The corresponding values for unstimulated cell cultures have been subtracted. Values from donors homozygous for HLA-DR15 were halved to obtain the MFI value per allele. *p*-values indicate probabilities for no difference between cytokine-producing B-cell subsets and the total B-cell pool (Wilcoxon matched-pairs signed rank test), or between study groups (two-tailed Mann Whitney U-test). NS: Not significant.

Given the ability of B cells to efficiently capture and present antigen [12], the B-cell cytokine responses shown here are likely to shape the function of encephalitogenic T cells. To examine the cytokine context in which T cells see presentation of MBP-derived peptides by B cells, we took advantage of the mAb MK16 recognizing MBP85-99 associated with HLA-DR15 [31]. As shown in Fig 5A–5D, bulk B cells from HLA-DR15+ RRMS patients and healthy donors did not differ with respect to their ability to present the immunodominant epitope MBP85-99 after stimulation with whole MBP.

IL-6-producing B cells generally presented MBP85-99 relatively poorly compared to the bulk of B cells. This was most pronounced in the healthy donor group, where almost no antigen presentation was observed in 4 out of 7 tested donors (Fig 5B).

IL-10-producing B cells from healthy donors, however, presented MBP85-99 as efficiently as the background B-cell population (Fig 5D), whereas IL-10-producing B cells from RRMS patients showed diminished capacity to present the peptide—both in comparison to the patients’ B-cell population as a whole and, with borderline significance, to IL-10-producing B cells from healthy donors.

Finally, we assessed the presentation of MBP85-99 in B cells stimulated with a combination of PMA + ionomycin and MBP. In general, the presentation of MBP85-99 was lowered after inclusion of PMA + ionomycin as inducer, and no differences were observed between RRMS patients and healthy donors (data not shown).

## Discussion

The promising effect of B-cell depletion therapy in patients with RRMS [6,7,9] demonstrates their important role in the pathogenesis of MS. The limited effect of such therapy on the production of autoantibodies suggests that B cells exert their pathogenic effect through secretion of cytokines or antigen presentation. In the present study, we investigated these functions of B cells from RRMS patients. Unlike previous studies using polyclonal B-cell stimulation with PMA, ionomycin, CpG, or BCR cross-linking, we stimulated PBMCs with the MS-relevant self-antigen MBP. Moreover, the presence during stimulation of autologous serum containing naturally occurring or disease-associated anti-MBP antibodies and an intact complement system distinguished our study from previous studies on B-cell cytokine production.

Our key finding is that MBP induced B cells producing TNF-α or IL-6 in PBMC cultures derived from RRMS patients, but only to a limited extent in cultures from healthy donors. Both TNF-α and IL-6 have been implicated in RRMS and EAE. At autopsy, MS patients have elevated TNF levels at the site of active MS lesions [34], and TNF-α levels in cerebrospinal fluid and serum correlate with the severity of the lesions [35]. With respect to IL-6, it has been shown that B cells from RRMS patients produce more of this cytokine than B cells from healthy donors [21]. Notably, repopulating B cells isolated from patients after B-cell depletion therapy showed normal IL-6 production [21]. Moreover, IL-6 has been shown to be essential for development of EAE [36–40]. Examining the effects of B-cell depletion in mice, Barr *et al*. showed that B cells account for 65–95% of the IL-6 production induced by LPS, CpG, or anti-CD40, and thus were the major source of IL-6 in secondary lymphoid tissues [21]. They demonstrated that B-cell depletion improved disease in IL-6-sufficient mice, but not in IL-6-deficient mice [21]. An important effect of IL-6 in EAE—and presumably in MS—is its ability to induce Th17 responses [24], which have a pathogenic role in autoimmune disease [41].

In parallel with MBP stimulation alone, we assessed B-cell cytokine production when PMA and ionomycin were added for the last four hours of incubation. When BCR signaling was thus bypassed, patients with RRMS and healthy donors displayed similar frequencies of B cells producing TNF-α or IL-6, suggesting that the differences described above for MBP-stimulated cells were antigen-specific. In analogy, Bar-Or and colleagues, using anti-IgG/IgM and co-culture with mouse fibroblasts transfected with CD40-ligand to mimic antigenic stimulation and T-cell help, showed that B cells from RRMS patients and healthy donors produce similar amounts of TNF-α and lymphotoxin, unless stimulated with either IFN-γ or pathogen-associated CpG-DNS as 3^rd^ signal [5,23]. In our experimental set-up, IFN-γ may have been produced by T cells present in the co-cultures, as previously shown in a similar setting [42].

In cultures stimulated with PMA + ionomycin and MBP, the frequencies of both IL-6-producing and TNF-α-producing B cells correlated with the EDSS score. Moreover, borderline-significant correlations with the MSSS score were also found. Thus, severe MS seems to be associated with relatively high frequencies of B cells producing IL-6 or TNF-α.

Over recent years, IL-10-producing B cells have been shown to protect against autoimmune diseases, including EAE [19,20]. Only few studies have used a similar approach to ours, i.e. stimulation with a disease-relevant self-antigen, for induction of IL-10 production by B cells [15,25,26]. Kristensen *et al*. found no difference between the frequencies of IL-10-producing B cells from patients with Graves’ disease or Hashimoto’s thyroiditis and those of healthy donors [26]. Concordantly, we found no difference between the frequency of MBP-induced IL-10-producing B cells in healthy donors and RRMS patients, as one group. We did, however, observe a decreased frequency of IL-10-producing B cells in the patients with the most severe disease in terms of EDSS and MSSS scores. Using polyclonal stimuli (various combinations of cross-linking of BCRs, CD40 engagement, CpG stimulation, and PMA + ionomycin stimulation) to maximize the frequency of cytokine-producing B cells, other groups have found significant decreases in the frequency of IL-10-producing B cells in patients with rheumatoid arthritis [43,44], Graves’ disease [45], and MS [5,23]. In discordance with these studies—and with the above-mentioned B-cell responses to MBP—we found a surprising positive correlation between EDSS and the frequency of IL-10-producing B cells after stimulation with PMA + ionomycin, which closely reflected the frequencies of B cells producing TNF-α or IL-6. We interpret this as an attempt by B cells to compensate for excessive production of pro-inflammatory cytokines, as described by Shen and Fillatreau [46].

It has been reasoned that B cells, irrespective of antigenic specificity, can be stimulated through toll-like receptors to produce cytokines, whereas only myelin-specific B cells present antigen to encephalitogenic T cells [21]. When analyzing the ability of B cells to present the immunodominant peptide MBP85-99 by means of the antibody MK16, we observed no differences in antigen-presenting capacity between the bulk of B cells from RRMS patient and healthy donors, which both displayed a shift in the entire B cell population to the right indicating that all B cells became engaged in antigen presentation. We recently demonstrated that complement is instrumental in the uptake of MBP via complement receptor 2 (CR2, CD21) and in subsequent presentation of MBP85-99 [15]. The mechanism by which complement is activated by MBP—and other self-antigens such as thyroglobulin (TG) [47]–remains to be elucidated, but the presence of MBP-reactive antibodies in normal human serum and in serum from MS patients [48,49] suggests that immune complexes containing MBP activate complement through the classical pathway leading to tagging of MBP with fragments of complement component 3. In this way, the majority of B cells can bind MBP via CR2 and become engaged in antigen presentation [15].

We observed that our patients’ IL-10-producing B cells presented fewer copies of MBP85-99 than the corresponding B cells from healthy donors. Moreover, in addition to being reduced in frequency as described above, IL-6-producing B cells presented hardly any MBP85-95 in 4 out of 7 healthy donors. HLA-DR may be downregulated during differentiation of cytokine-producing B cells into plasma cells, but this can be expected to occur in patient cells and cells from healthy donors. This finding therefore underlines that B-cell presentation of self-antigen peptides is accompanied by cytokine production skewed towards a pro-inflammatory profile in RRMS. This may contribute to the shaping of an encephalitogenic T-cell response. In support of this notion, B-cell depletion therapy with rituximab in MS leads to a reduced number of T cells in the CSF [11], diminished production of IFN-γ and IL-17, and proliferation of peripheral T cells [5]. However, rituximab may deplete CD20dim T cells from the peripheral circulation of patients with MS [50], so the effects of rituximab on T cells are not necessarily mediated by B-cell depletion.

Taken together, the findings presented here suggest that B cells from RRMS patients are skewed toward a pro-inflammatory profile following stimulation with the self-antigen MBP. After polyclonal stimulation of patient B cells, the frequency of IL-6-producing and TNF-α-producing B cells correlated positively with disease severity. Importantly, in patients with the most severe disease, the ability of B cells to produce IL-10 after stimulation with MBP only was much diminished, suggesting compromised immunoregulatory function of self-reactive B cells in these patients.

## Supporting Information

S1 Fig Strategy for analysis of cytokine-producing B cells. Mononuclear cells were left unstimulated (-Stim, left column), or stimulated with whole MBP (middle column) or a combination of MBP for 24 hours and PMA + ionomycin for the last 4 hours of incubation (right column). LIVE/DEAD^®^ stain was used to discriminate living cells from dead cells, and doublets were excluded based on FSC-A and FSC-W. B cells were identified as CD19 positive cells within a morphological lymphocyte gate, and B cells producing (A) TNF-α, (B) IL-6, or (C) IL-10 were gated as shown for one representative RRMS patient.
(TIF)

S2 Fig Frequencies of B cells producing TNF-α, IL-6, or IL-10. Mononuclear cells from healthy donors (HD; N = 12) and patients with relapsing-remitting multiple sclerosis (RRMS; N = 13) were either left unstimulated (-stim), or stimulated with whole MBP for 24 hours (+MBP) or with MBP for 24 hours and PMA + ionomycin for the last 4 hours of incubation (+MBP+PMAiono). Cells were stained intracellularly with antibodies against (A) TNF-α, (B) IL-6 and (C) IL-10 before assessment by flow cytometry. The raw data corresponding to Fig 1 are shown as median, interquartile range (box) and range (whiskers). *p-*values indicate probabilities of no difference between groups (two-tailed Mann Whitney U-test) or between different treatments (Wilcoxon matched-pairs signed rank test). NS: Not significant.
(TIF)
