# Supplementary figures and images for: Myelin Basic Protein-Induced Production of Tumor Necrosis Factor-α and Interleukin-6, and Presentation of the Immunodominant Peptide MBP85-99 by B Cells from Patients with Relapsing-Remitting Multiple Sclerosis
Source: PLoS One. 2016 Jan 12;11(1):e0146971. doi: 10.1371/journal.pone.0146971 (PMC4710535; doi:10.1371/journal.pone.0146971)

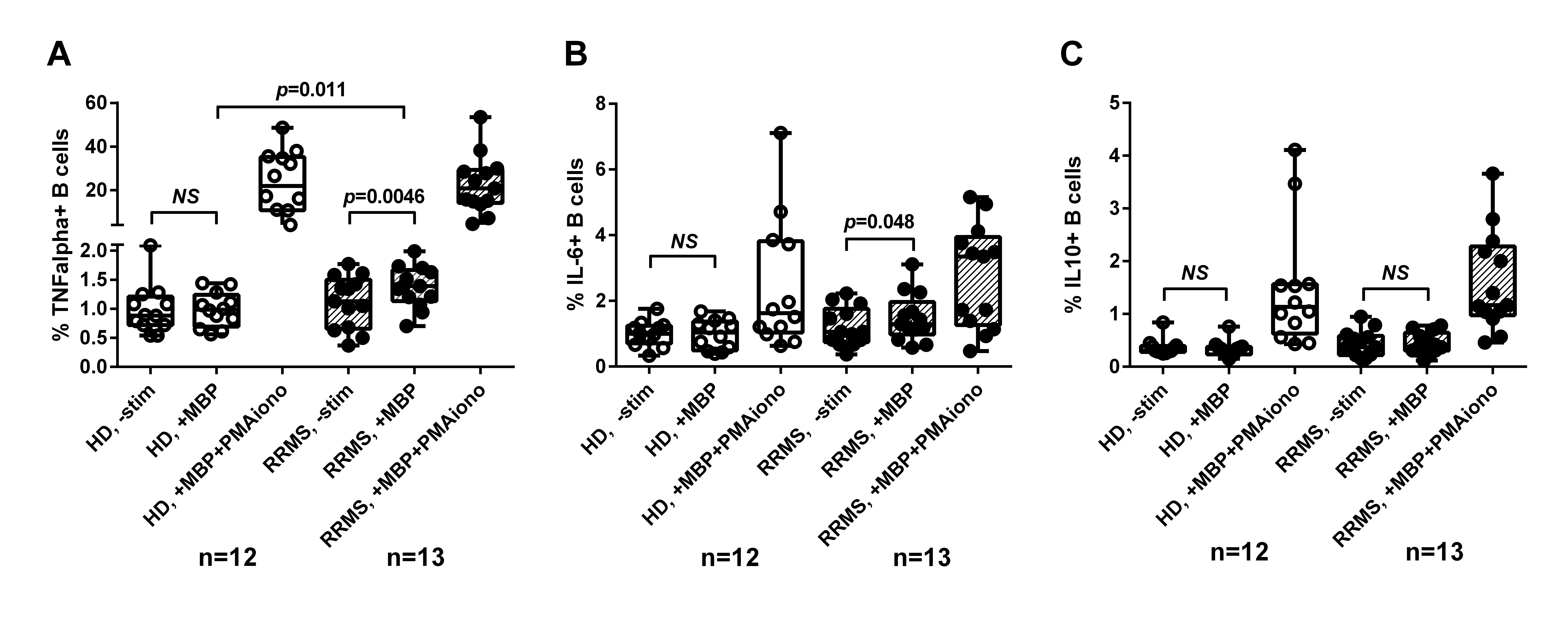

Supplement: S2 Fig — Mononuclear cells from healthy donors (HD; N = 12) and patients with relapsing-remitting multiple sclerosis (RRMS; N = 13) were either left unstimulated (-stim), or stimulated with whole MBP for 24 hours (+MBP) or with MBP for 24 hours and PMA + ionomycin for the last 4 hours of incubation (+MBP+PMAiono). Cells were stained intracellularly with antibodies against (A) TNF-α, (B) IL-6 and (C) IL-10 before assessment by flow cytometry. The raw data corresponding to Fig 1 are shown as median, interquartile range (box) and range (whiskers). p-values indicate probabilities of no difference between groups (two-tailed Mann Whitney U-test) or between different treatments (Wilcoxon matched-pairs signed rank test). NS: Not significant. (TIF) [file pone.0146971.s002.tif]
